# Luminescent conjugated oligothiophenes distinguish between α-synuclein assemblies of Parkinson’s disease and multiple system atrophy

**DOI:** 10.1186/s40478-019-0840-1

**Published:** 2019-12-03

**Authors:** Therése Klingstedt, Bernardino Ghetti, Janice L. Holton, Helen Ling, K. Peter R. Nilsson, Michel Goedert

**Affiliations:** 1 MRC Laboratory of Molecular Biology, Cambridge, CB2 0QH UK; 2 Department of Pathology and Laboratory Medicine, Indiana University School of Medicine, Indianapolis, IN 46202 USA; 3 Queen Square Brain Bank, UCL Queen Square Institute of Neurology, London, WC1N 1PJ UK; 4 Department of Physics, Chemistry and Biology, Linköping University, 581 83 Linköping, Sweden

**Keywords:** α-Synuclein, Luminescent conjugated oligothiophene, Multiple system atrophy, Neurodegeneration, Parkinson’s disease, Spectral analysis

## Abstract

Synucleinopathies [Parkinson’s disease with or without dementia, dementia with Lewy bodies and multiple system atrophy] are neurodegenerative diseases that are defined by the presence of filamentous α-synuclein inclusions. We investigated the ability of luminescent conjugated oligothiophenes to stain the inclusions of Parkinson’s disease and multiple system atrophy. They stained the Lewy pathology of Parkinson’s disease and the glial cytoplasmic inclusions of multiple system atrophy. Spectral analysis of HS-68-stained inclusions showed a red shift in multiple system atrophy, but the difference with Parkinson’s disease was not significant. However, when inclusions were double-labelled for HS-68 and an antibody specific for α-synuclein phosphorylated at S129, they could be distinguished based on colour shifts with blue designated for Parkinson’s disease and red for multiple system atrophy. The inclusions of Parkinson’s disease and multiple system atrophy could also be distinguished using fluorescence lifetime imaging. These findings are consistent with the presence of distinct conformers of assembled α-synuclein in Parkinson’s disease and multiple system atrophy.

## Introduction

Accumulation of α-synuclein (α-syn) assemblies is the pathological hallmark of a group of neurodegenerative diseases called synucleinopathies [1]. Parkinson’s disease (PD) is the most common synucleinopathy and the second most common neurodegenerative disease, after Alzheimer’s disease (AD). Diagnosis requires bradykinesia, in conjunction with at least one of the following signs: rigidity, tremor and postural instability. However, post mortem examination of the brain is required to firmly establish diagnosis [2]. Neuropathologically, PD is characterised by dopaminergic nerve cell loss in the *pars compacta* of the substantia nigra and the presence of filamentous α-syn assemblies in the form of Lewy bodies (LBs) and Lewy neurites (LNs). The α-syn of LBs and LNs [3] is post-translationally modified, with phosphorylation of S129 (pS129) being the most prominent modification [4, 5]. While only approximately 4% of α-syn is phosphorylated at S129 in normal brain, more than 90% of assembled α-syn carries this modification [5]. Therefore, antibodies directed against pS129 are often used to identify α-syn deposits in PD and other synucleinopathies.

Assemblies of α-syn are also characteristic of Lewy body dementia (LBD) and multiple system atrophy (MSA) [6–8]. LBD encompasses cases of dementia with Lewy bodies (DLB) and Parkinson’s disease dementia (PDD) [1, 9]. MSA includes cases of olivopontocerebellar atrophy, striatonigral degeneration and mixed MSA as determined by the pattern of neurodegeneration on examination of post mortem brain tissue [10, 11]. LBD presents predominantly as a progressive dementia with varying degrees of motor involvement, whereas MSA is characterized by a combination of parkinsonian, cerebellar and autonomic symptoms. In MSA, α-syn aggregates are present in both nerve cells and glial cells, chiefly oligodendrocytes, where they form glial cytoplasmic inclusions (GCIs) [6–8, 11]. α-Syn filaments isolated from the brains of MSA patients can have different morphologies from those extracted from the brains of patients with PD and DLB [6, 12–15]. Moreover, LBs and GCIs demonstrate different abilities to seed α-syn aggregation in cell culture and in mouse models [15–19]. These results have led to the hypothesis that aggregated α-syn has different conformations in PD and MSA brains, a phenomenon that has previously been established for assembled tau in Alzheimer’s and Pick’s diseases by electron cryo-microscopy [20, 21].

Luminescent conjugated oligothiophenes (LCOs) are fluorescent ligands that detect protein aggregates in human diseases and models thereof [22]. Solid-state nuclear magnetic resonance has shown that LCOs bind in grooves along the filament axis of HET-s aggregates, where they interact with charged amino acids [23]. LCOs detect a larger spectrum of aggregates than amyloid ligands, such as Congo red and thioflavins [24, 25]. The colour of light emitted from a given LCO is determined by the conformation of its flexible thiophene backbone, which in turn depends on the conformations of the assemblies it binds to. Thus, distinct conformers of assembled proteins can be separated based on the colour of the LCO, and this has provided new information about prion and Aβ strains [26, 27].

Here we show that LCOs can be used to detect α-syn assemblies in brain from patients with PD and MSA. We also show that, in combination with labelling of pS129 α-syn, they provide evidence for the existence of distinct conformers of assembled α-syn.

## Materials and methods

### LCO staining

Frozen brain tissues from neuropathologically confirmed cases of PD and MSA, as well as healthy controls, were obtained from the Queen Square Brain Bank and the Dementia Laboratory at the Department of Pathology and Laboratory Medicine, Indiana University School of Medicine. Brain regions were substantia nigra and/or cingulate gyrus for PD, cerebellum for MSA, and cerebellum and midbrain at the level of substantia nigra for healthy controls. See Table 1 for additional information. The synthesis of HS-68 has been described [28]. Frozen brain sections (10 μm) were fixed in 96% ethanol for 10 min, rehydrated and incubated in phosphate-buffered saline (PBS) for 10 min. HS-68 (3 μM in PBS) was added for 30 min at room temperature. The sections were then washed in PBS and mounted (Dako). The mounting medium was allowed to solidify for approximately 20 h before the samples were analysed. Sections were also stained with LCOs p-FTAA and h-FTAA. Syntheses of p-FTAA and h-FTAA have been described [24, 29].

**Table 1:** Description of cases

Case no	Diagnosis	Brain region	Age/Sex
1	MSA	Cbl	75/F
2	MSA	Cbl	82/M
3	MSA	Cbl	68/F
4	MSA	Cbl	69/M
5	MSA	Cbl	65/F
6	MSA	Cbl	71/F
7	MSA	Cbl	83/F
8	MSA	Cbl	52/M
9	PD	Sn/Cg	74/F
10	PD	Sn/Cg	92/M
11	PD	Sn/Cg	64/M
12	PD	Sn/Cg	83/M
13	PD	Sn	74/M
14	PD	Sn	79/M
15	PD	Sn	86/F
16	PD	Sn	75/M
17	PD	Cg	92/F
18	Control	Cbl	82/F
19	Control	Cbl	56/F
20	Control	Cbl	80/M
21	Control	Cbl/Mb-Sn	69/M
22	Control	Cbl/Mb-Sn	74/M

*Cbl* cerebellum, *Cg* cingulate gyrus, *Mb* midbrain, *MSA* multiple system atrophy, *PD* Parkinson’s disease, *Sn* substantia nigra

#### Immunohistochemistry

Frozen brain sections (10 μm) were fixed in acetone at − 20 °C for 5 min, allowed to dry for 30 min and rehydrated in PBS for 1 min. They were then incubated in PBS with 5% normal goat serum (blocking buffer) for 30 min at room temperature. A rabbit monoclonal antibody specific for pS129 α-syn (ab51253, Abcam) was used at 1:1000 in blocking buffer. After 2 h at room temperature, the sections were washed with PBS for 3 × 5 min and signal visualized with a goat anti-rabbit antibody conjugated to Alexa 488 fluorophore (Thermo Fisher Scientific) diluted 1:400 in blocking buffer. After 1 h at room temperature, the sections were washed with PBS for 3 × 5 min and mounted. Fluorescence images were collected using an inverted Zeiss LSM 780 confocal microscope.

### Double-labelling

Brain sections were stained with anti-pS129 α-syn as described above, with the exception that goat anti-rabbit secondary antibody conjugated with Alexa 647 (Thermo Fisher Scientific) was used (1:200 in blocking buffer) to avoid interference with LCO emission. PD sections were also stained with anti-α-syn antibody (Syn303, 1:1000, Biolegend), anti-phosphorylated tau antibody (AT100, 1:250, Thermo Fisher Scientific), anti-p62 antibody (1:100, BD Bioscience) and anti-TDP-43 antibody (1:500, Proteintech Group Inc.). After washing 3 × 5 min in PBS, they were incubated with 3 μM HS-68 for 30 min at room temperature. They were then washed with PBS and mounted with Dako mounting medium. Fluorescence images and emission spectra were collected using an inverted Zeiss LSM 780 confocal microscope.

### Spectral analysis

Spectral analysis was performed on frozen brain sections fixed with ethanol and stained with HS-68, and on sections fixed with acetone and double-labelled with HS-68 and anti-pS129 α-syn antibody. Emission spectra were collected from HS-68-labelled structures using an excitation wavelength of 405 nm. For pS129-positive α-syn aggregates labelled with HS-68, an excitation wavelength of 458 nm was used. Spectral analysis was performed on an inverted Zeiss LSM 780 confocal microscope. In ethanol-fixed sections, the HS-68 spectrum from 1 to 10 regions of interest per aggregate was collected from at least 15 aggregates per sample. However, for samples 11, 9* and 11*, the number of analysed aggregates was less. In the HS-68 and pS129 co-labelling spectral experiment, 3 PD and 3 MSA cases were included. The number of analysed double labelled aggregates was 29 or more, each containing 2–14 regions of interest.

### Fluorescence lifetime imaging

Sections were stained with pS129 α-syn antibody and h-FTAA as described above, and fluorescence lifetime imaging of stained tissue sections was acquired on an inverted Zeiss LSM 780 confocal microscope equipped with a 32 channel QUASAR GaAsP spectral array detector. Emitted photons were routed through the direct coupling confocal port of the Zeiss LSM 780 scanning unit and detected by a Becker & Hickl HPM-100-40 hybrid photomultiplier tube (Becker & Hickl GmbH). Data were recorded by a Simple-Tau 152 system (SPC-150 TCSPC FLIM module) with the instrument recording software SPCM version 9.42 in the FIFO image mode using 256 time-channels. A Plan-Apochromat 40×/1.3 Oil DIC objective lens was used, and the pinhole set to 20.2 μm. For excitation at 490 nm, a pulsed tunable In Tune laser with a repetition rate of 40 MHz was used. Analysis used SPCImage version 3.9.4; 17–44 α-syn aggregates from 3 PD and 3 MSA cases were included.

### Statistics

Each spectrum represents the mean emission for a number of regions of interest collected from either a single aggregate or a group of aggregates. For HS-68, the plots show the ratio of fluorescence intensity for each region of interest at emission wavelengths of 485 or 486 and 573 nm (485/573_R_, 486/573_R_). The standard deviation (S.D.) for each sample is included in the ratio plot graph. To determine if there was a significant difference in LCO emission between α-syn aggregates in PD and MSA, a Mann-Whitney test (GraphPad Prism 6) was performed.

## Results

### LCO staining

**Fig. 1:**
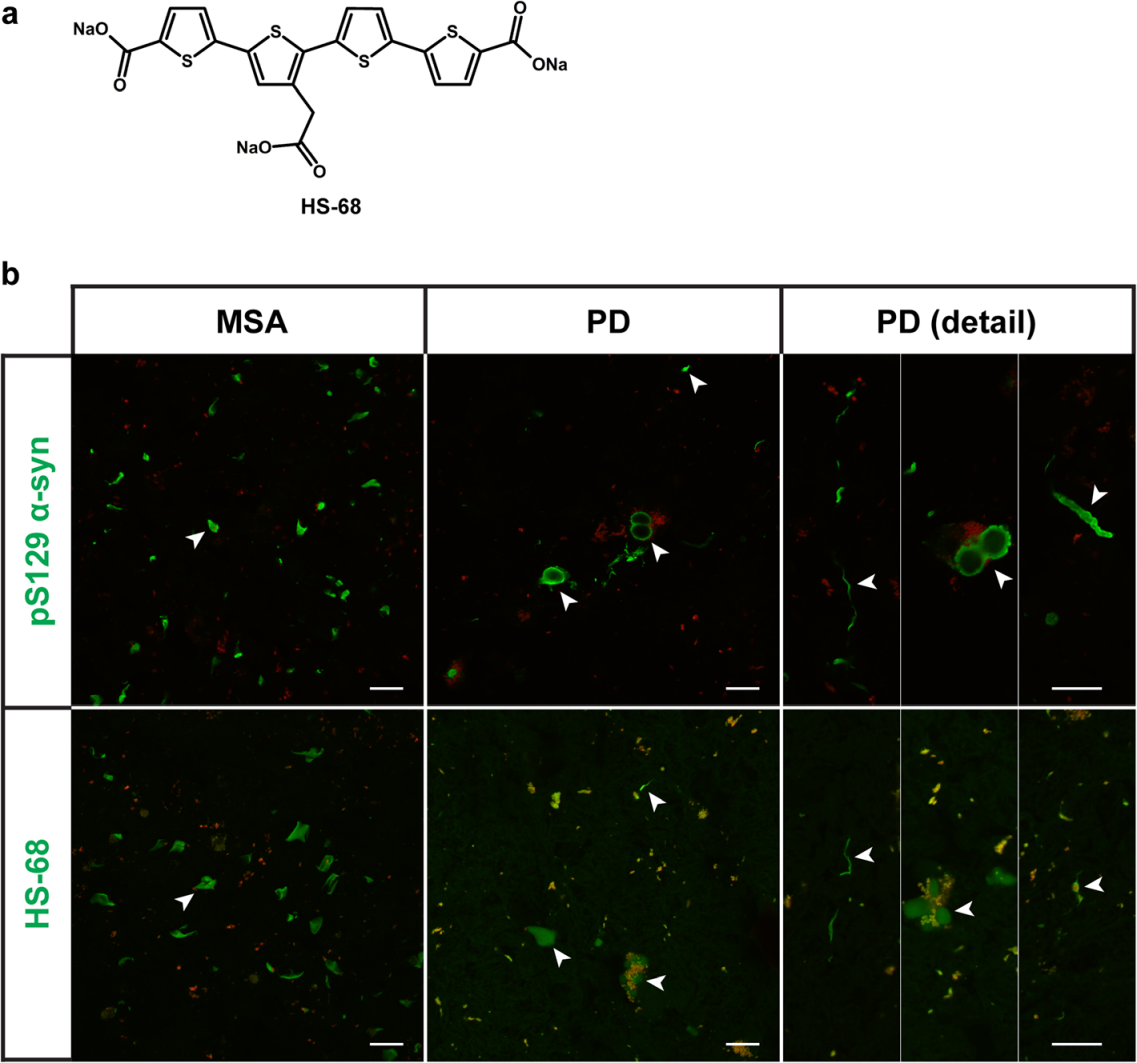
HS-68-positive inclusions in multiple system atrophy (MSA) and Parkinson’s disease (PD). **a** Structure of the luminescent conjugated oligothiophene HS-68. **b** MSA and PD brain sections from cases 8 (cerebellum) and 15 (substantia nigra), respectively, stained with HS-68 and labelled using the pS129 α-syn antibody. HS-68 stained glial cytoplasmic inclusions in MSA (arrowheads), as well as Lewy bodies and Lewy neurites in PD (arrowheads). Red/orange structures represent autofluorescent lipofuscin. Scale bars, 20 μm.

Brain sections from patients with MSA and PD were stained with HS-68 (Fig. 1a). Staining was compared with that of an antibody specific for pS129 α-syn. In MSA, the pS129 antibody labelled GCIs, which varied in size and appearance, being sickle-, flame- or ghost-shaped, and HS-68 displayed a similar staining pattern (Fig. 1b). In PD, pS129-positive LBs appeared as cytoplasmic spheres and, occasionally, tangle-like assemblies. LNs and neuropil threads were also seen. All α-syn deposits identified by the pS129 antibody were stained by HS-68. Staining of LBs with HS-68 was more homogenous than with the pS129 antibody which, in most cases, only labelled the LB periphery (Fig. 1b). In PD, HS-68 staining was weaker for a small number of aggregates than for the majority of inclusions.

### Spectral analysis of inclusions stained by HS-68

**Fig. 2:**
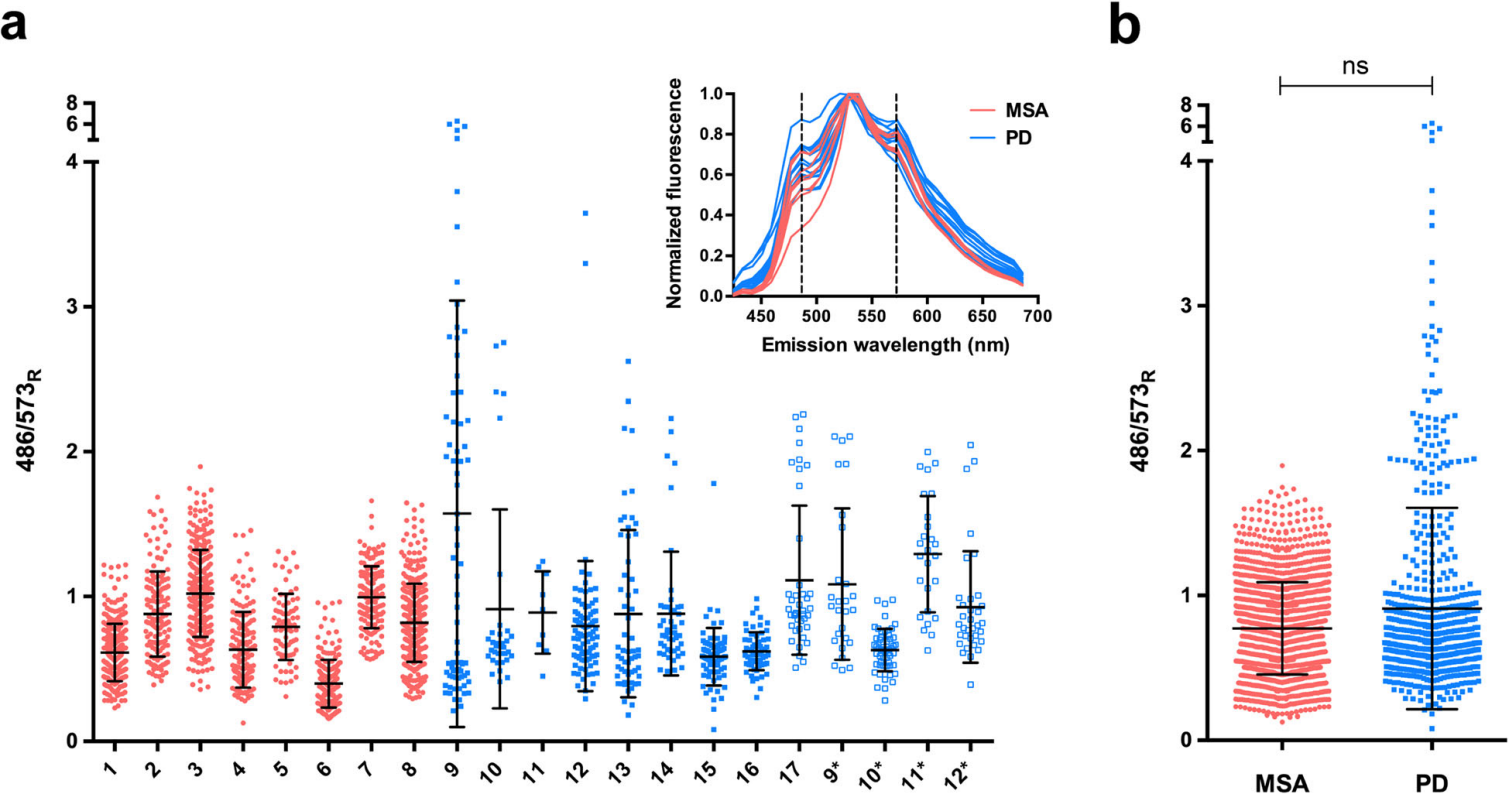
Spectral analysis of HS-68-positive inclusions in multiple system atrophy (MSA) and Parkinson’s disease (PD). **a** Plot of the ratio of emission intensities at wavelengths of 486 and 573 nm for HS-68 following binding to inclusions in MSA (coral circles) and PD (cyan squares). Cerebellum was used for MSA; substantia nigra (filled squares) and cingulate gyrus (open squares) for PD. Each dot corresponds to a region of interest. The 8 cases of MSA (1–8) and 9 cases of PD (9–17) are listed in Table 1. Asterisk indicates cases for which both substantia nigra and cingulate gyrus were used. Insert: Mean HS-68 emission spectra when binding to inclusions in brain tissues from MSA (coral) and PD (cyan). Each spectrum represents one MSA (cerebellum) or one PD (substantia nigra and/or cingulate gyrus) case. Dashed lines indicate the wavelengths that were used for calculating the ratio of emission intensity depicted in the plots. **b** Merging of the ratio values for all MSA (coral circles) and PD (cyan squares) cases shown in **a**. The results are expressed as means ± S.D.; ns, not significant.

**Fig. 3:**
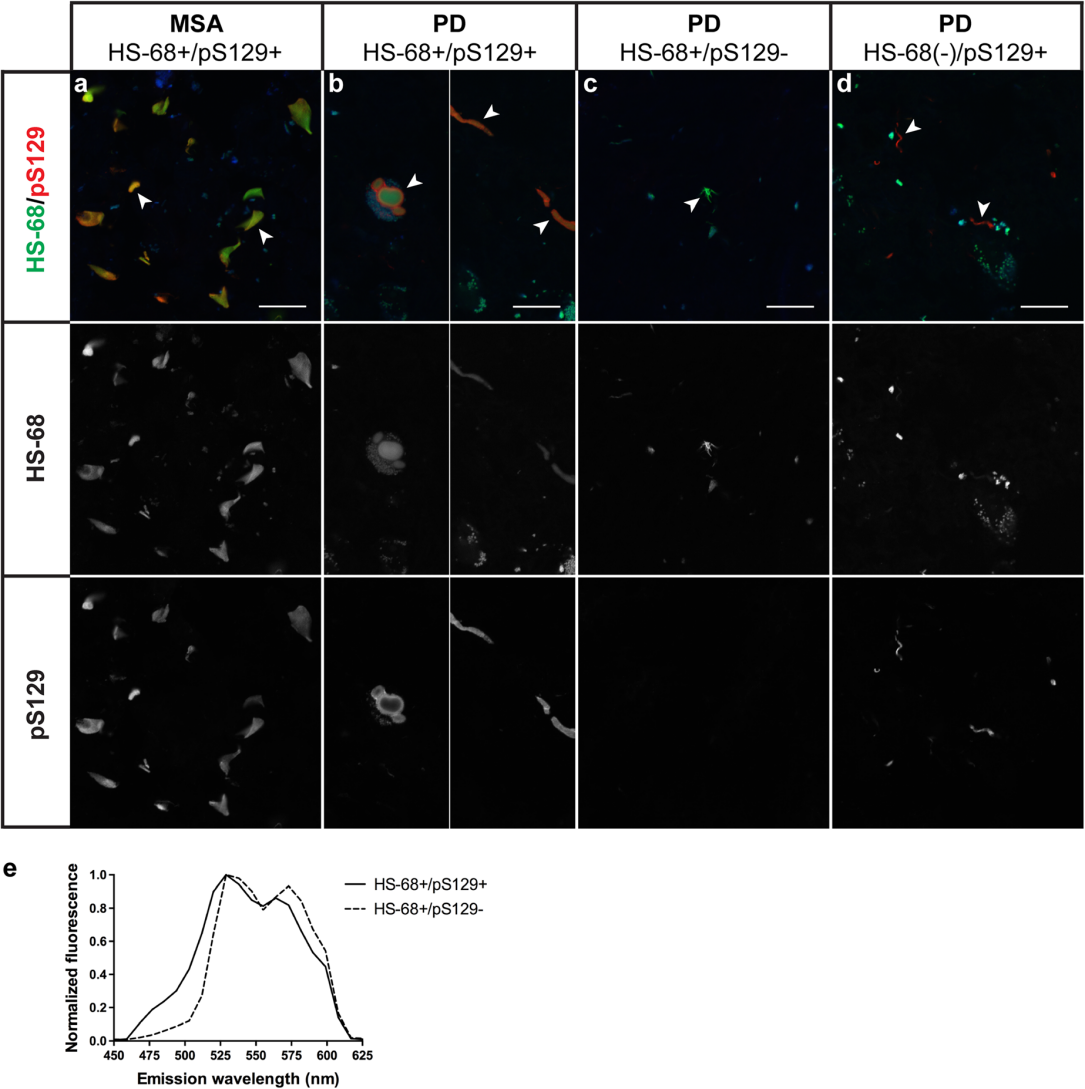
HS-68- and pS129 α-syn antibody-positive inclusions in multiple system atrophy (MSA) and Parkinson’s disease (PD). **a** Fluorescence image of brain tissue section from MSA case 8 (cerebellum) stained with HS-68 and labelled with pS129 α-syn antibody. GCIs (arrowheads) showed complete co-localization between HS-68 and pS129 antibody. **b-d** Section from PD case 15 (substantia nigra) stained with HS-68 and pS129 α-syn antibody, showing aggregates positive for HS-68 and pS129 (arrowheads) **(b);** HS-68-positive, pS129-negative, aggregates (arrowhead) **(c);** weakly HS-68-positive, pS129-positive, aggregates (arrowheads) **(d)**. Channels for single staining are shown in white to enhance visualization. Blue/turquoise structures represent autofluorescent lipofuscin, which is also visible in the channel for HS-68 fluorescence. **e** Mean HS-68 emission spectra collected from HS-68-positive, pS129-positive (solid line) and HS-68-positive, pS129-negative (dashed line) aggregates indicated by arrowheads in b and c, respectively. A representative experiment is shown. Similar results were obtained using 2 additional cases of each PD and MSA. Scale bars, 20 μm

Emission spectra of HS-68 varied within and between cases of MSA and PD (Fig. 2). They were further analysed by calculating the ratios of fluorescence intensity at wavelengths with the most pronounced variation. Most cases of MSA displayed a lower mean than PD cases, indicating a red shift of emission (Fig. 2a, b), but the difference was not statistically significant (Fig. 2b). However, in PD samples stained with HS-68, emission spectra divided aggregates into two groups: a red-shifted cluster that overlapped with MSA and a blue-shifted cluster that did not overlap (Fig. 2a**)**. Blue-shifted aggregates included LBs, whereas red-shifted aggregates were smaller (Fig. 3). For some PD cases, spectral analysis was performed on protein inclusions from both substantia nigra and cingulate gyrus (Table 1). Although the means were not identical, the spectral trends were similar (Fig. 2a). However, in cingulate gyrus, the cluster of aggregates that did not spectrally overlap with MSA showed a less pronounced blue-shift compared with the corresponding cluster in substantia nigra. We also used LCOs p-FTAA and h-FTAA for spectral analysis [24, 29]. Similar to HS-68, they bound to inclusions identified by the pS129 α-syn antibody. However, HS-68 was better at identifying shifts in colour between MSA and PD.

### Double-labelling (HS-68 and pS129 α-syn antibody)

In MSA cerebellar white matter, co-localisation between HS-68 and the pS129 α-syn antibody was observed (Fig. 3a). The labelling intensity of the antibody varied, with some GCIs showing a weaker signal. In PD, two types of aggregate were present: i) HS-68-positive, pS129-positive; ii) HS-68-positive, pS129-negative (Fig. 3b, c). Fluorescence intensity varied in the first group, with a small number of aggregates only displaying weak HS-68 staining (Fig. 3d). Only the pS129-positive inclusions were blue-shifted (Fig. 3e). The pS129-negative species, which were smaller than LBs, represented the red-shifted cluster of aggregates (Fig. 2a). Labelling with Syn303, AT100, anti-p62 and anti-TDP-43 antibodies showed no co-localisation with HS-68 in these structures. It therefore remains to be determined what the constituents of the red-shifted HS-68-positive and pS129-negative aggregates in PD are. In general, when performing double-labelling, the HS-68 emission spectrum was red-shifted, with the shoulder at 573 nm being more pronounced and that at 485 nm almost abolished (Fig. 3e), compared to when only HS-68 was used (Fig. 2a). This was probably because another fixative (acetone instead of ethanol) and a different excitation wavelength were used. No co-labelling of HS-68 and pS129 α-syn antibody could be seen in the control cases.

### Spectral analysis of double-labelled aggregates

**Fig. 4:**
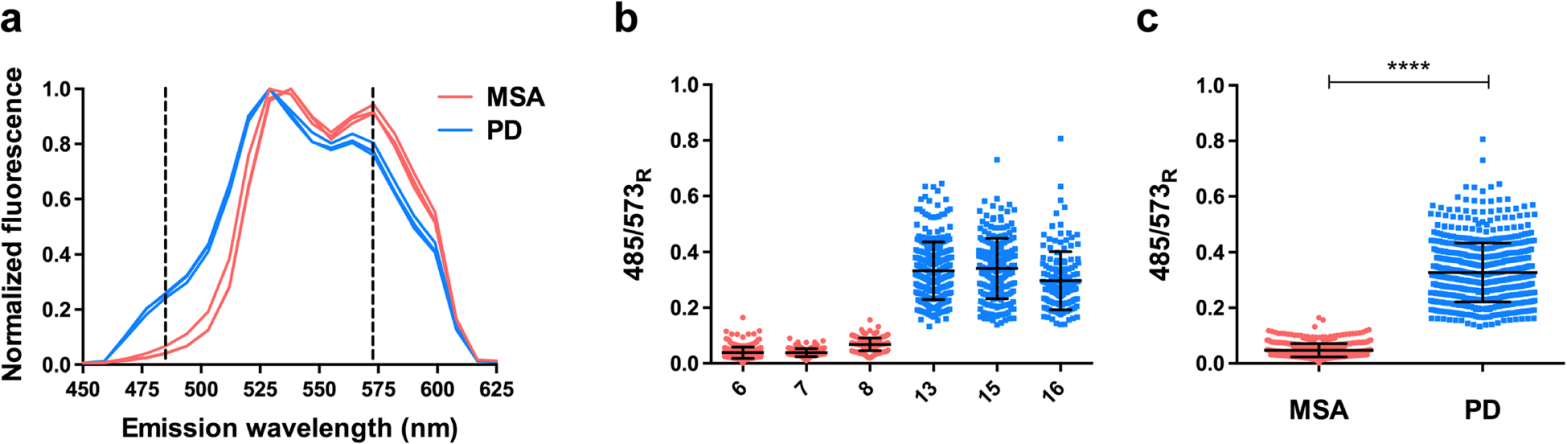
Spectral analysis of HS-68- and pS129 α-syn antibody-positive inclusions in multiple system atrophy (MSA) and Parkinson’s disease (PD). **a** Mean emission spectra of HS-68 binding to pS129 α-syn positive deposits in MSA cases 6, 7 and 8 (coral, cerebellum) and PD cases 13, 15 and 16 (cyan, substantia nigra). **b** Plot of the ratio of emission intensity at wavelengths 485 and 573 nm for HS-68 when binding to inclusions in MSA (coral circles) and PD (cyan squares) that were also labelled with pS129 α-syn antibody. **c** Merging of the ratio values for all MSA (coral circles) and PD (cyan squares) cases shown in b. The results are expressed as means ± S.D. (*n* = 3) ****p < 0.0001

A second spectral analysis was performed that included only aggregates labelled by both HS-68 and the pS129 α-syn antibody. HS-68 emission graphs gave a similar maximum at 529 nm (PD) and/or 538 nm (MSA) (Fig. 4a); however, the overall contour of the spectra revealed a shift in colour. In PD sections, there was a distinct emission shoulder at 485 nm, which was not present in MSA (Fig. 4a). Instead, the dominating shoulder in MSA was at 573 nm. These differences show that pS129 α-syn assemblies in PD were blue-shifted relative to MSA. To assess the results in more detail, and to visualize the spectral distribution of each sample, the ratios of fluorescence intensity at 485 nm and 573 nm (485/573_R_) were calculated. PD samples showed a higher mean than MSA cases, confirming the blue-shift in emission (Fig. 4b). Spectral differences were significant (Fig. 4c) and showed that HS-68 can be used to separate the α-syn aggregates of PD and MSA. The shift in colour indicates that HS-68 binds differently to α-syn assemblies in PD and MSA, suggesting that they are characterised by different assembled α-syn conformers.

### Fluorescence lifetime imaging of h-FTAA-stained, pS129-labelled α-syn aggregates

**Fig. 5:**
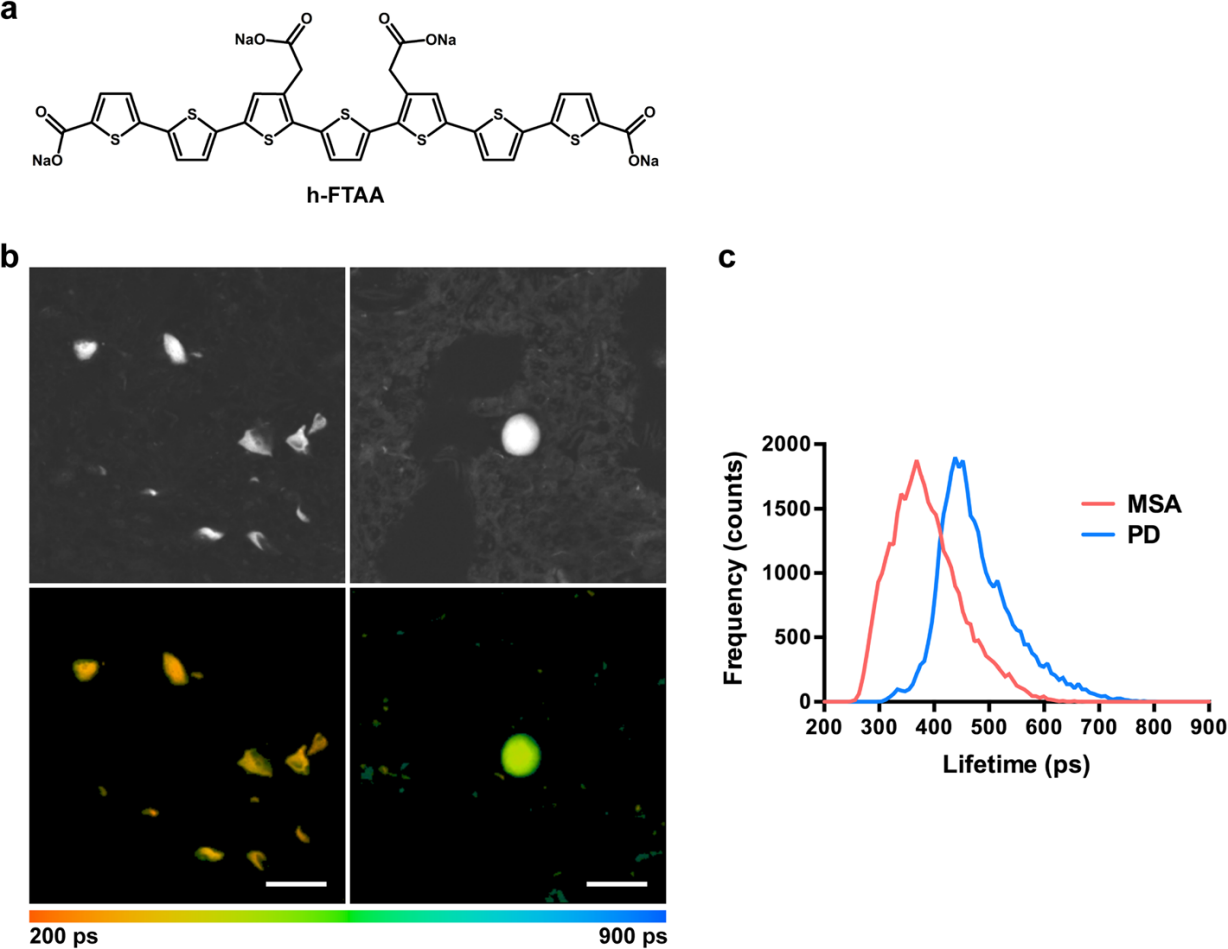
Fluorescence lifetime imaging of h-FTAA- and pS129 α-syn antibody-positive inclusions in multiple system atrophy (MSA) and Parkinson’s disease (PD). **a** Structure of the luminescent conjugated oligothiophene h-FTAA**. b** Fluorescence intensity (top) and fluorescence lifetime (bottom) images of h-FTAA stained pS129 positive α-syn inclusions in MSA (case 6, cerebellum) and PD (case 13, substantia nigra) brains. The colour bar represents lifetimes from 200 ps (orange)-900 ps (blue) and the images are colour coded according to lifetime. **c** Fluorescence lifetime distributions of h-FTAA bound to pS129 positive α-syn deposits in MSA (coral line) and PD (cyan line); 17–44 inclusions from 3 cases of MSA (6, 7 and 8, cerebellum) and 3 cases of PD (13, 15 and 16, substantia nigra) were used. Scale bar, 20 μm

Measuring the decay of light emitted from a bound LCO shows how it binds, since the fluorescence decay of a fluorophore is sensitive to its conformation and the surrounding chemical environment [30]. Fluorescence lifetime imaging (FLIM) microscopy using h-FTAA (Fig. 5a) has previously revealed polymorphisms of prion aggregates and Aβ plaques [31, 32]. We therefore investigated the fluorescence lifetime of h-FTAA binding to pS129-positive aggregates in PD and MSA. h-FTAA showed a fluorescence decay of 300–700 ps in PD and of 250–600 ps in MSA (Fig. 5). Thus, a distinct distribution of fluorescence decay was observed, indicating that h-FTAA binds in different ways to the binding pockets of PD and MSA aggregates. No co-labelling of h-FTAA and pS129 α-syn antibody could be seen in the control cases.

## Discussion

LCOs have been used to study protein aggregates in human diseases and experimental models thereof [22]. Here we show that HS-68 can detect α-syn assemblies in brain sections from patients affected by synucleinopathy. The ligand showed strong emission when binding to GCIs in MSA and various types of α-syn inclusions in PD, and gave similar staining to that of an antibody specific for pS129 α-syn. However, when binding to LBs, the LCO displayed more homogenous staining than the antibody. Most brainstem LBs consist of a dense core surrounded by a corona of radiating filaments [33]. HS-68 stained both core and corona, whereas the antibody labelled mostly the corona. The smaller size of HS-68 may have allowed better penetration. HS-68 exhibited a greater variation in fluorescence intensity in PD than in MSA. One explanation may be a greater diversity of aggregates. Two types of LBs have been described previously, with core and corona being more typical of brainstem than cortical LBs [34]. Different shapes of α-syn assemblies are also seen in neuronal processes such as thread-like LNs and spheroid structures. Alternatively, HS-68 may bind to the weakly fluorescent aggregates in a more quenched manner. Like thioflavin T [35, 36], HS-68 may have to be sterically locked in the binding pocket to become highly fluorescent.

Spectral properties of LCOs are conformation-dependent. Structural analyses, as well as seeding experiments in cells and animals, have suggested that α-syn aggregates may show conformational variation [6, 12–19]. Spectral analysis of HS-68 showed a red-shift for MSA aggregates, but the difference with PD was not significant. However, when HS-68 and pS129 α-syn antibody double-labelled aggregates were analysed, the α-syn deposits of PD and MSA were distinguishable, because of their blue and red colours. Since the emission profiles of LCOs are dependent on conformation [22], it follows that α-syn assemblies in PD and MSA are conformationally distinct.

Differences in the cellular environment may underlie the formation of distinct α-syn conformers in PD and MSA. Thus, it has been reported that oligodendrocytes, but not neurons, can convert α-syn into a conformer that is like that of GCIs [16]. Moreover, injection of PD and MSA brain extracts into mice transgenic for A53T α-syn showed that the silver staining properties of induced neuronal assemblies of α-syn were dependent on both transgene expression and cellular environment [37]. The largest shifts in the HS-68 emission spectrum for α-syn assemblies of PD and MSA were present at wavelengths of 486 nm and 573 nm, respectively. Previously, intensity shifts were found in the same region when Aβ plaques and tau inclusions in young and old transgenic mice were compared [38]. Aβ and tau deposits showed a shift from red to blue in HS-68 emission, as the mice were ageing. Since spectral analysis showed that the α-syn assemblies of PD were blue-shifted relative to those of MSA, it is possible that the inclusions of PD were more mature. Also, in PD, the blue-shift was more pronounced for inclusions in substantia nigra, which is a region targeted early in the disease progression, compared with inclusions in later involved cingulate gyrus [39]. It has been reported that α-syn assemblies from PD brains are less detergent-soluble than those from MSA brains [13]. Moreover, PD is diagnosed at a similar age as MSA, but it has in general a significantly longer duration [10]. It remains to be seen if LCOs can distinguish between the α-syn inclusions of atypical cases of PD and MSA with similar durations.

To characterise PD aggregates stained by HS-68, but not labelled with the pS129 α-syn antibody, additional co-labelling was performed. However, the identity of the component(s) of these inclusions could not be identified. Further analysis, perhaps combining laser capture microdissection and mass spectrometry or the use of epitope-specific α-syn antibodies, will be needed. An alternative explanation for the lack of co-labelling could be that antibody and LCO competed for the same binding sites, as described for prions [27].

We also performed FLIM of h-FTAA and pS129 antibody-labelled α-syn deposits in brain tissue sections. The lifetime of a fluorophore, i.e. the time it spends in the excited state before returning to the ground state, is dependent on its conformation and on how it interacts with its environment [30]. One example is the difference in fluorescence decay exhibited by h-FTAA when binding to distinct prion conformers [31]. FLIM analysis of h-FTAA has also been used to distinguish Aβ morphotypes associated with ApoE deficiency in APPPS1 mice [32]. We now show that the fluorescence decay of h-FTAA differed when binding to α-syn aggregates in PD compared to MSA. Hence, similar to the spectral result, FLIM demonstrated that LCOs can interact in different ways with α-syn deposits in PD and MSA.

In conclusion, we show that LCOs can be used to detect accumulations of α-syn in PD and MSA. Moreover, by analysing LCO staining, we show that pS129 positive α-syn aggregates in PD differ from those in MSA regarding ligand stainability, emission profiles and fluorescence lifetimes. It follows that distinct conformations of assembled α-syn are present in the brains of patients with PD and MSA. Structural analysis of α-syn filaments from PD and MSA brains is required to prove the existence of distinct conformers and identify their differences.

## Abbreviations

AD: Alzheimer’s disease
α-syn: α-synuclein.
DLB: Dementia with Lewy bodies
FLIM: Fluorescence lifetime imaging
GCI: Glial cytoplasmic inclusion
LB: Lewy body
LBD: Lewy body dementia
LCO: Luminescent conjugated oligothiophene
LN: Lewy neurite
MSA: Multiple system atrophy
PBS: Phosphate buffered saline
PD: Parkinson’s disease
PDD: Parkinson’s disease dementia
pS129: phosphorylated serine at position 129
